# Clinical characteristics of patients treated with immune checkpoint inhibitors in *EGFR*-mutant non-small cell lung cancer: CS-Lung-003 prospective observational registry study

**DOI:** 10.1007/s00432-024-05618-4

**Published:** 2024-02-12

**Authors:** Tadahiro Kuribayashi, Kadoaki Ohashi, Kazuya Nishii, Kiichiro Ninomiya, Yukari Tsubata, Nobuhisa Ishikawa, Masahiro Kodani, Nobuhiro Kanaji, Masahiro Yamasaki, Kazunori Fujitaka, Shoichi Kuyama, Nagio Takigawa, Nobukazu Fujimoto, Tetsuya Kubota, Masaaki Inoue, Keiichi Fujiwara, Shingo Harita, Ichiro Takata, Kenji Takada, Sachi Okawa, Katsuyuki Kiura, Katsuyuki Hotta

**Affiliations:** 1 Department of Hematology, Oncology and Respiratory Medicine, Okayama University Graduate School of Medicine, Dentistry and Pharmaceutical Sciences, Okayama, Japan; 2 Department of Allergy and Respiratory Medicine, Okayama University Hospital, Okayama, Japan; 3 Department of Internal Medicine, Division of Medical Oncology and Respiratory Medicine, Faculty of Medicine, Shimane University, Izumo, Japan; 4 Department of Respiratory Medicine, Hiroshima Prefectural Hospital, Hiroshima, Japan; 5 Division of Respiratory Medicine and Rheumatology, Department of Multidisciplinary Internal Medicine, Faculty of Medicine, Tottori University, Yonago, Japan; 6 Department of Internal Medicine, Division of Hematology, Rheumatology, and Respiratory Medicine, Faculty of Medicine, Kagawa University, Miki, Kagawa Japan; 7 Department of Respiratory Medicine, Hiroshima Red Cross Hospital and Atomic-Bomb Survivors Hospital, Hiroshima, Japan; 8 Department of Molecular and Internal Medicine, Graduate School of Biomedical and Health Sciences, Hiroshima University, Hiroshima, Japan; 9 Department of Respiratory Medicine, National Hospital Organization Iwakuni Clinical Center, Iwakuni, Japan; 10 Department of Internal Medicine 4, Kawasaki Medical School, Okayama, Japan; 11 Department of Medical Oncology, Okayama Rosai Hospital, Okayama, Japan; 12 Department of Respiratory Medicine and Allergology, Kochi University Hospital, Kochi, Japan; 13 Department of Chest Surgery, Shimonoseki City Hospital, Shimonoseki, Japan; 14 Department of Respiratory Medicine, NHO Okayama Medical Center, Okayama, Japan; 15 Department of Internal Medicine, Okayama Saiseikai General Hospital, Okayama, Japan; 16 Internal Medicine, Fukuyama City Hospital, Fukuyama, Japan; 17 Internal Medicine, Kajiki Hospital, Okayama, Japan

**Keywords:** EGFR, EGFR-TKI, Lung cancer, Immune checkpoint inhibitors, Performance status

## Abstract

**Purpose:**

Immune checkpoint inhibitors (ICIs) are ineffective against *epidermal growth factor receptor* (*EGFR*)-mutant non-small cell lung cancer (NSCLC). This study aimed to investigate the clinical characteristics of patients who were treated or not treated with ICIs, and of those who benefit from immunotherapy in *EGFR*-mutant NSCLC.

**Methods:**

We analyzed patients with unresectable stage III/IV or recurrent NSCLC harboring *EGFR* mutations using a prospective umbrella-type lung cancer registry (CS-Lung-003).

**Results:**

A total of 303 patients who met the eligibility criteria were analyzed. The median age was 69 years; 116 patients were male, 289 had adenocarcinoma, 273 had major mutations, and 67 were treated with ICIs. The duration of EGFR-TKI treatment was longer in the Non-ICI group than in the ICI group (17.1 vs. 12.7 months, *p* < 0.001). Patients who received ICIs for more than 6 months were categorized into the durable clinical benefit (DCB) group (24 patients), and those who received ICIs for less than 6 months into the Non-DCB group (43 patients). The overall survival in the DCB group exhibited longer than the Non-DCB group (69.3 vs. 47.1 months), and an equivalent compared to that in the Non-ICI group (69.3 vs. 68.9 months). Multivariate analysis for time to next treatment (TTNT) of ICIs showed that a poor PS was associated with a shorter TTNT [hazard ratio (HR) 3.309; *p* < 0.001]. Patients who were treated with ICIs and chemotherapy combination were associated with a longer TTNT (HR 0.389; *p* = 0.003). In addition, minor *EGFR* mutation was associated with a long TTNT (HR 0.450; *p* = 0.046).

**Conclusion:**

ICIs were administered to only 22% of patients with *EGFR*-mutated lung cancer, and they had shorter TTNT of EGFR-TKI compared to other patients. ICI treatment should be avoided in *EGFR* mutated lung cancer with poor PS but can be considered for lung cancer with *EGFR* minor mutations. Pathological biomarker to predict long-term responders to ICI are needed.

**Supplementary Information:**

The online version contains supplementary material available at 10.1007/s00432-024-05618-4.

## Introduction

Lung cancer is the leading cause of cancer-related deaths worldwide (Sung et al. 2021). *Epidermal growth factor receptor (EGFR)* mutations account for 50–60% of driver oncogenes of lung adenocarcinomas in individuals of the East Asian ethnicity or never smokers (Shigematsu H et al. 2005). EGFR tyrosine kinase inhibitors (EGFR-TKIs) provide a survival benefit in *EGFR*-mutant non-small-cell lung cancer (NSCLC) (Ohashi et al. 2013; Soria et al. 2018). However, the inhibitory effect of EGFR-TKIs is transient and disease progression is inevitable owing to the acquisition of resistance (Passaro et al. 2021).

Immune checkpoint inhibitors (ICIs) such as programmed cell death-1 and anti-programmed death-ligand 1 (PD-L1) inhibitors prolong the overall survival (OS) of patients with lung cancer (Ferrara et al. 2021; Zhou et al. 2020). However, they have a limited effect on *EGFR*-mutant NSCLCs (Lee et al. 2018). In contrast, ICIs occasionally exert sustained tumor inhibition in some *EGFR*-mutant lung cancers (Garassino et al. 2018; Watanabe et al. 2019). The characteristics of patients who may benefit from ICIs have not yet been fully established. Therefore, we aimed to compare the clinical characteristics of ICI-treated and non-treated patients with *EGFR*-mutant lung cancers and to investigate the characteristics of those who benefited from immunotherapy in a prospective registry cohort of NSCLC.

## Materials and methods

### Patients and study design

This observational study was registered at the prospective umbrella-type lung cancer registry (CS-Lung-003; UMIN000026696) (Nishii et al. 2021; Kudo et al. 2022) and included patients with lung cancer enrolled from 31 collaborating hospitals between July 2017 and September 2020. This study aimed to investigate clinical practice patterns and treatment efficacy in patients with *EGFR*-mutant lung cancer. Data for this study were collected in August 2021. This study was approved by the ethics committee of the participating hospital (no. 1703–055; Institutional Review Board of Okayama University Hospital) and all patients provided written informed consent.

### Patient eligibility

This study included patients with unresectable stage III/IV lung cancer harboring *EGFR* mutations without indications for radical radiotherapy or with recurrent *EGFR*-mutant NSCLC. We excluded patients with an observation period of less than 6 months or unknown outcome. *EGFR* mutations were assessed using a test approved by the Pharmaceuticals and Medical Devices Agency of Japan. We defined the *EGFR* exon 19 deletion and *EGFR* L858R as major mutations, and the other types as minor mutations. We categorized patients treated with ICIs for more than 6 months in the “durable clinical benefit (DCB)” group, and those treated with ICIs for less than 6 months in the “Non-DCB” group (Rizvi NA et al. 2015). Patients treated with ICIs for more than 2 years were considered as “long-term responders” (von Pawel J et al. 2019).

### Outcomes

The primary outcome was the OS, compared between the ICI and Non-ICI groups. Secondary outcomes were the frequency of ICI use in *EGFR* lung cancer, OS in the DCB and Non-DCB groups, and clinical factors correlated with time to next treatment (TTNT) for immunotherapy. TTNT was calculated from the date of initiation of EGFR-TKI or ICI therapy to the date of next treatment or death due to any cause (Kehl et al. 2021). OS was calculated from the date of initiation of first-line anti-cancer therapy to the date of death or the last follow-up.

### Statistical analyses

Patient characteristics were assessed using Fisher’s exact test. The Kaplan–Meier method was used for analysis of TTNT and OS. TTNT for EGFR-TKI or ICI therapy and OS were assessed using the log-rank test. Univariate and multivariate analyses were performed using a Cox proportional hazards model to evaluate the factors associated with the duration of ICI treatment. A multivariate analysis was conducted using the stepwise method, with threshold *p* values for entering and removing variables (Eastern Cooperative Oncology Group performance status (PS), histology, type of *EGFR* mutation, treatment, sex, age, smoking history, and line of treatment) from the model as 0.05 and 0.20, respectively. All statistical analyses were performed using STATA software (version 17.0) (StataCorp, College Station, Texas, USA) and *p* values < 0.05 were considered statistically significant.

## Results

### Efficacy of EGFR-TKIs in *EGFR*-mutant lung cancer with/without immunotherapy

**Fig. 1:**
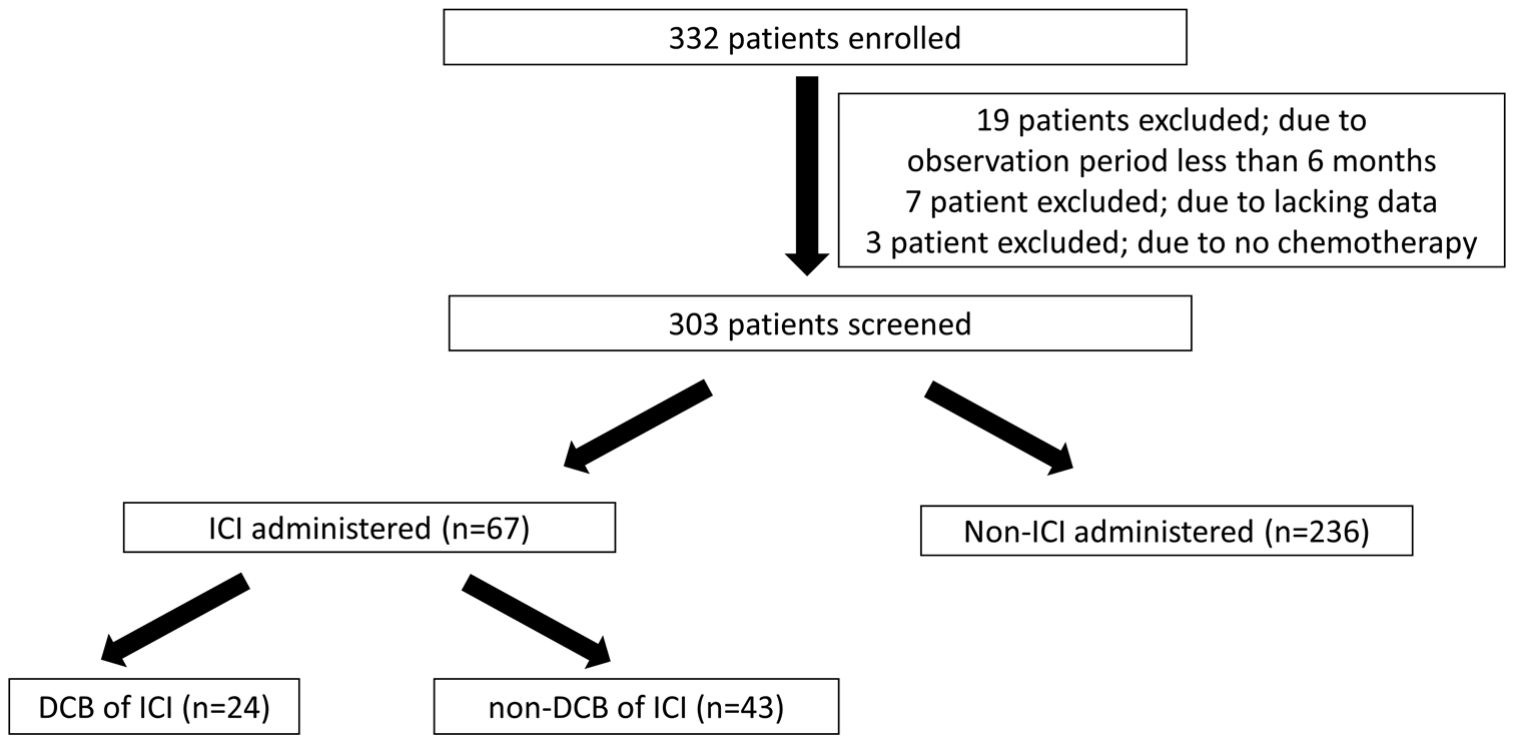
Study flow chart

A total of 332 patients with stage III/IV disease harboring *EGFR* mutations without an indication for radical radiotherapy or surgery or with *EGFR*-mutant recurrent NSCLC were consecutively enrolled in this registry study from July 2017 to September 2020 (Fig. 1). Of these, 29 patients were excluded because of a short observation period (less than 6 months) (*n* = 19), lack of data (*n* = 7), or no therapy (*n* = 3). Of the remaining 303 patients, 67 (22%) were treated with ICIs, and 236 (78%) were not.

**Fig. 2:**
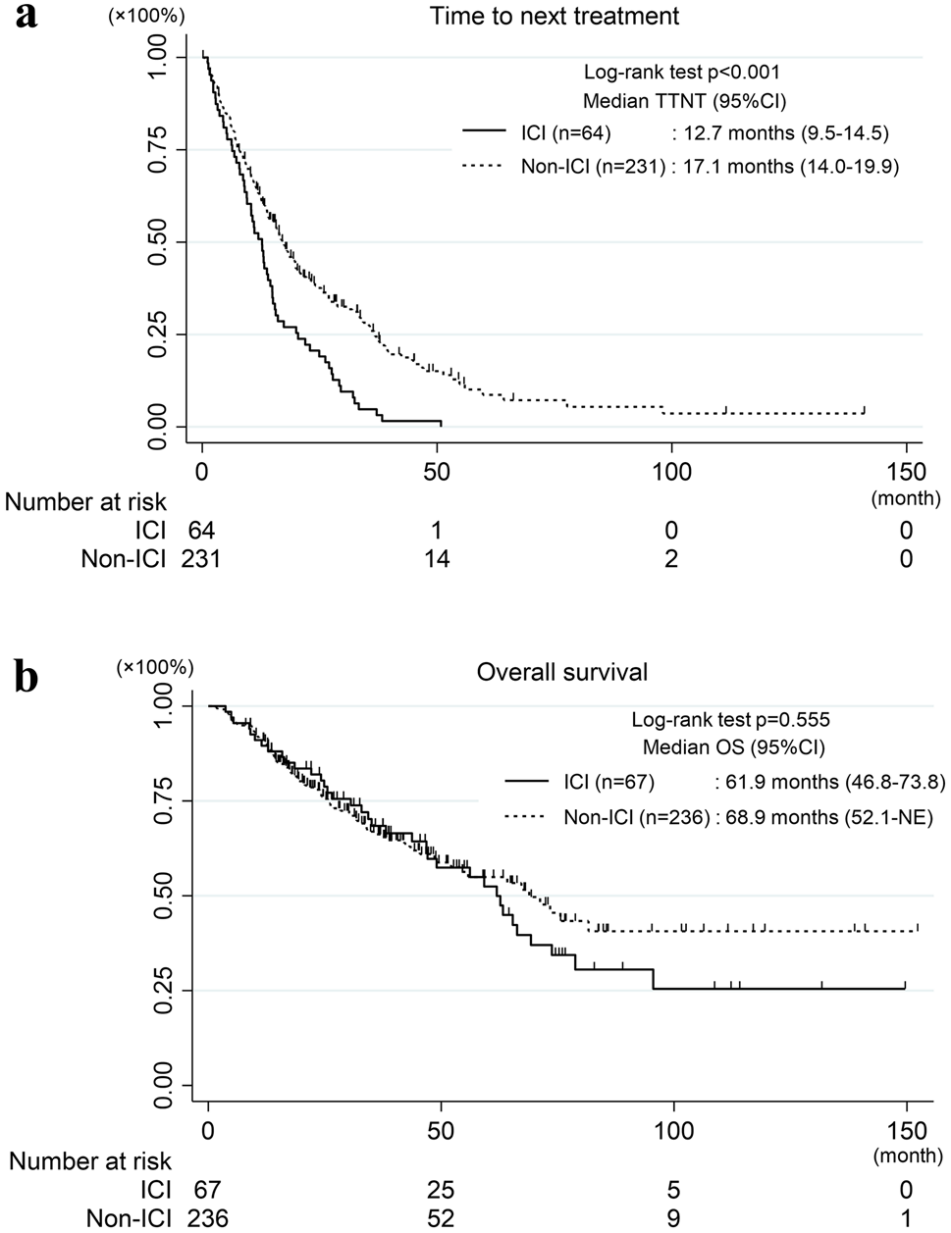
Kaplan–Meier curves for TTNT for EGFR-TKI (**a**) and OS in patients with ICI and Non-ICI treatment (**b**)

First, we analyzed the clinical characteristics of 303 patients (Table 1). The median patient age was 69 (range 26–98) years. Of the included patients, 38% were men, 59% were non-smokers, 95% had adenocarcinoma, and 84% had PS 0–1 at the initiation of systemic therapy. *EGFR* exon 19 deletion was observed in 55%, exon 21 L858R in 35%, and minor mutations in the remaining 10% patients. Initial use of EGFR-TKIs was as follows: the 1st generation (gefitinib and erlotinib) were used in 40% patients, 2nd generation (afatinib) in 33%, and 3rd generation (osimertinib) in 24%. 3% of all patients had never been treated with EGFR-TKIs. Then, we evaluated patient characteristics according to the provision of ICI treatment (Table 2). Compared with those in the ICI group, patients in the Non-ICI group were significantly more likely to be female and have brain metastases at diagnosis. There were no significant differences between age, the groups in stage, histology, PS, *EGFR* mutation type, liver metastasis, or smoking history. In addition, 4% in the ICI group and 2% in the non-ICI group never received EGFR-TKIs (*p* = 0.381). The duration of EGFR-TKI treatment was significantly shorter in the ICI group than in the Non-ICI group (median 12.7 vs. 17.1 months, *p* < 0.001) (Fig. 2a). Given that osimertinib showed superior effect than gefitinib or erlotinib (Soria et al. 2018), we excluded patients who were treated with osimertinib as initial EGFR-TKI (Supplementary Table 1), and assessed the TTNT of EGFR-TKI in patients treated with 1st or 2nd generation EGFR-TKIs as initial EGFR-TKI. The TTNT were still shorter in the ICI group than in the Non-ICI group (median 12.8 vs. 18.5 months, *p* < 0.001) (Supplementary Fig. 1a). In contrast, patients in the Non-ICI group tended to have a longer OS than those in the ICI group, in patients with or without osimertinib treatment, although the difference was not significant (median 68.9 vs. 61.9 months, *p* = 0.555) (Fig. 2b) (median 75.6 vs. 63.3 months, *p* = 0.364) (Supplementary Fig. 1b). Consequently, these results suggest that the duration of EGFR-TKI treatment is more important than the administration of ICI for a survival benefit in patients with *EGFR* mutated lung cancers.

**Table 1:** Patient characteristics (*n* = 303)

Median age, years (range)	69 (26–98)
Sex (male/female)	116 (38%)/187 (62%)
Stage (III, IV/recurrent)	235 (78%)/68 (22%)
PS at the initiation of systemic therapy (0–1/2–4/unknown)	256 (84%)/36 (12%)/11 (4%)
Histology (Ad/others)	289 (95%)/14 (5%)
*EGFR* mutation type (19 del or L858R/others)	273 (90%)/30 (10%)
Metastasis of brain (yes/no)	68 (22%)/235 (78%)
Metastasis of liver (yes/no)	23 (8%)/280 (92%)
Generations of EGFR-TKI (1st/2nd/3rd/IND/never)	121 (40%)/99 (33%)/73 (24%)/2 (1%)/8 (3%)
Smoking history (yes/no/unknown)	119 (39%)/180 (59%)/4 (1%)

*PS* performance status, *Ad* adenocarcinoma, *EGFR* epidermal growth factor receptor, *19 del* exon 19 deletion, *L858R* exon 21 L858R point mutation, *TKI* tyrosine kinase inhibitor, *IND* Investigational new drug

**Table 2:** Patient characteristics of ICI and Non-ICI groups

	ICI (*n* = 67)	Non-ICI (*n* = 236)	*p* value
Median age, years (range)	64 (26–91)	70 (36–98)	
Age (≥ 75 years/ < 75 years)	13 (19%)/54 (81%)	74 (31%)/162 (69%)	0.066
Sex (male/female)	33 (49%)/34 (51%)	83 (35%)/153 (65%)	0.046
Stage (III, IV/recurrent)	48 (72%)/19 (28%)	187 (79%)/49 (21%)	0.189
Histology (Ad/others)	64 (96%)/3 (4%)	225 (95%)/11 (5%)	1.000
PS at the initiation of systemic therapy (0–1/2–4)	60 (90%)/4 (6%)	196 (83%)/32 (14%)	0.130
*EGFR* mutation type (19 del or L858R/others)	58 (87%)/9 (13%)	215 (91%)/21 (9%)	0.352
Metastasis of brain (yes/no)	9 (13%)/58 (87%)	59 (25%)/177 (75%)	0.047
Metastasis of liver (yes/no)	5 (7%)/62 (93%)	18 (8%)/218 (92%)	1.000
Smoking history (yes/no)	32 (48%)/33 (49%)	87 (37%)/147 (62%)	0.087
Generations of EGFR-TKI (1st/2nd/3rd/IND)	25 (37%)/29 (43%)/10 (15%)/0 (0%)	96 (41%)/70 (30%)/63 (27%)/2 (1%)	
Generations of EGFR-TKI (1st, 2nd, or IND/3rd)	54 (80%)/10 (15%)	168 (71%)/63 (27%)	0.071

*ICI* immune checkpoint inhibitor, *Ad* adenocarcinoma, *PS* performance status, *EGFR* epidermal growth factor receptor, *19 del* exon 19 deletion, *L858R* exon 21 L858R point mutation, *TKI* tyrosine kinase inhibitor, *IND* Investigational new drug

### Superior OS in the DCB than in the Non-DCB group in *EGFR*-mutant lung cancers

To examine the characteristics of patients who benefited from ICI treatment, we compared the DCB and Non-DCB groups. The DCB group included 36% (24/67) and the Non-DCB group included 64% (43/67) of patients treated with ICIs. The characteristics of each group are presented in Table 3. Comparing the DCB and Non-DCB groups, the DCB group had a better PS at ICI initiation, an earlier line of treatment (≤ third line), and a higher proportion of patients treated with chemotherapy and ICIs combination (ChemoIO). In contrast, there were no significant differences in age, sex, stage, histology, PS at the initiation of systemic therapy, type of *EGFR* mutation, smoking history, duration of EGFR-TKI treatment, or immune-related adverse events. The expression of PD-L1 was not examined in 71% of patients in the DCB group and 51% of those in the Non-DCB group. The types of minor *EGFR* mutations are as shown in Supplementary Table 2. For ChemoIO, the atezolizumab, bevacizumab, carboplatin and paclitaxel (ABCP) combination was the most commonly used (DCB group: 82%, Non-DCB group: 75%).

**Table 3:** Patient characteristics of DCB and Non-DCB groups

	DCB (n = 24)	Non-DCB (n = 43)	*p* value
Median age, years (range)	61 (29–84)	66(26–91)	
Age (≥ 75 years/ < 75 years)	3 (13%)/21 (88%)	10 (23%)/33 (77%)	0.350
Sex (male/female)	13 (54%)/11 (46%)	20 (47%)/23 (53%)	0.615
Stage (III, IV/recurrent)	16 (67%)/8 (33%) 33%)	32 (74%)/11 (26%)	0.576
Histology (Ad/Sq)	22 (92%)/2 (8%)	42 (98%)/1 (2%)	0.290
PS at the initiation of systemic therapy (0–1/2–4)	23 (96%)/0 (0%)	37 (86%)/4 (9%)	0.288
PS at ICI initiation (0–1/2–4)	23 (96%)/1 (4%)	26 (60%)/14 (33%)	0.005
*EGFR* mutation type (19 del or L858R/others)	19 (79%)/5 (21%)	39 (91%)/4 (9%)	0.264
PD-L1 TPS (< 1/1–49/ ≥ 50/unknown)	2 (8%)/2 (8%)/3 (13%)/17 (71%)	9 (21%)/7 (16%)/5 (12%)/22 (51%)	
PD-L1 TPS (< 1, 1–49, or unknown/ ≥ 50)	21 (88%)/3 (13%)	38 (88%)/5 (12%)	1.000
Line of treatment (≤ third line/ ≥ fourth line)	18 (75%)/6 (25%)	17 (40%)/26 (60%)	0.010
Treatment (ICI monotherapy/ICI + Chemotherapy)	7 (29%)/17 (71%)	35 (81%)/8 (19%)	< 0.001
Ate/Nivo/Pemb	2 (29%)/4 (57%)/1 (14%)	14 (40%)/10 (29%)/11 (31%)	
ABCP/CBDCA + PEM + Ate/CBDCA + PEM + Pemb	14 (82%)/2 (12%)/1 (6%)	6 (75%)/1 (13%)/1 (13%)	
Generations of EGFR-TKI (1st/2nd/3rd)	8 (33%)/12 (50%)/2 (8%)	17 (40%)/17 (40%)/8 (19%)	
Generations of EGFR-TKI (1st or 2nd /3rd)	20 (83%)/2 (8%)	34 (79%)/8 (19%)	0.472
Duration of EGFR-TKI, months (95% CI)	10.4 (7.2–15.6)	13.0 (9.5–14.9)	0.867
Smoking history (yes/no)	12 (50%)/11 (46%)	20 (47%)/22 (51%)	0.798
irAE (yes/no)	4 (17%)/19 (79%)	2 (5%)/39 (91%)	0.177

*DCB* durable clinical benefit, *Ad* adenocarcinoma, *Sq* squamous cell carcinoma, *PS* performance status, *ICI* immune checkpoint inhibitor, *EGFR* epidermal growth factor receptor, *19 del* exon 19 deletion, *L858R* exon 21 L858R point mutation, *PD-L1* programmed death-ligand 1, *TPS* Tumor proportion score, *Ate* atezolizumab, *Nivo* nivolumab, *Pemb* Pembrolizumab, *ABCP* Atezolizumab plus bevacizumab plus carboplatin and paclitaxel, *CBDCA* carboplatin, *PEM* pemetrexed, *TKI* tyrosine kinase inhibitor, *CI* confidence interval, *irAE* immune related adverse events

**Fig. 3:**
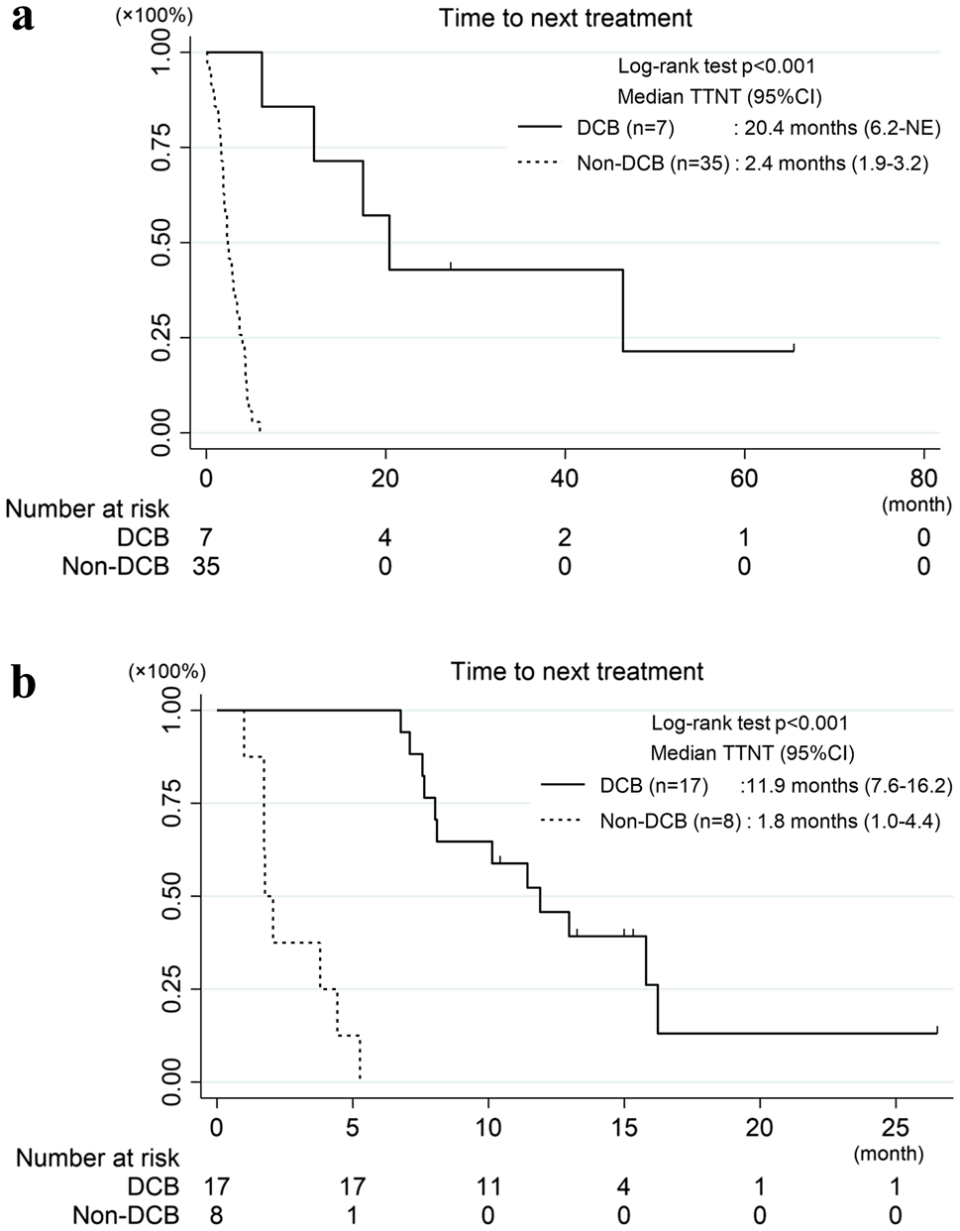
Kaplan–Meier curves for TTNT for ICI monotherapy (**a**) and ChemoIO (**b**) in the DCB and Non-DCB groups

**Fig. 4:**
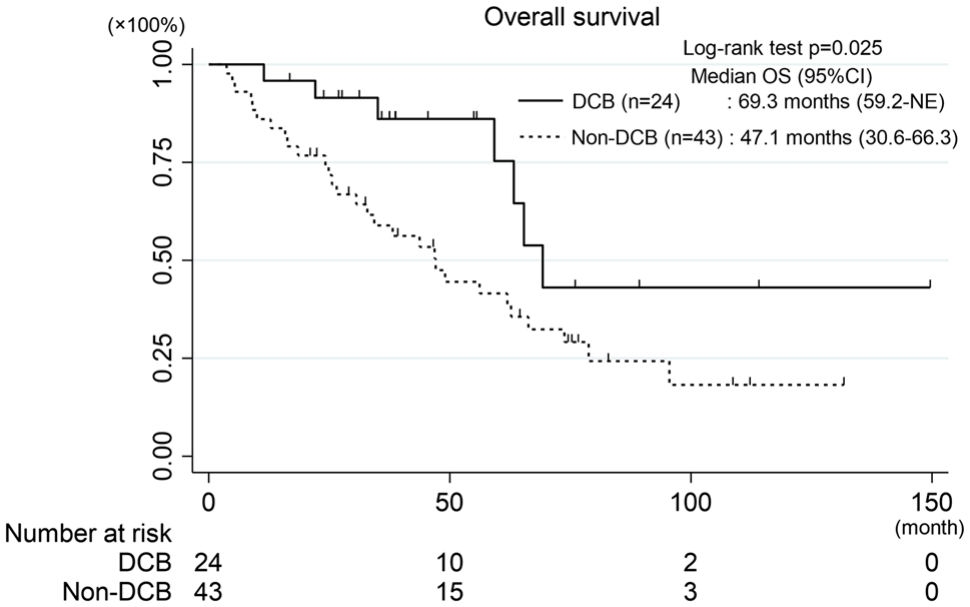
Kaplan–Meier curves for OS in the DCB and Non-DCB groups. *TTNT* time to next treatment, *EGFR* Epidermal growth factor receptor, *TKI* tyrosine kinase inhibitor, *OS* overall survival, *ICI* immune-checkpoint inhibitors, *ChemoIO* chemotherapy and ICIs combination, *DCB* durable clinical benefit, *CI* confidence interval, *NE* not evaluable

The TTNT for ICI monotherapy was 20.4 months in the DCB group and 2.4 months in the Non-DCB group (*p* < 0.001) (Fig. 3a). The TTNT for ChemoIO was 11.9 or 1.8 months in the DCB or Non-DCB group, respectively (*p* < 0.001) (Fig. 3b). The median OS was significantly longer in the DCB group than in the Non-DCB group (69.3 months vs. 47.1 months, *p* = 0.025) (Fig. 4) and was comparable to the Non-ICI group (Supplementary Fig. 2).

### Impact of PS, ChemoIO, minor mutilations on TTNT for immunotherapy in *EGFR*-mutant lung cancers

Univariate and multivariate analyses were performed in 67 patients to assess the clinical factors associated with TTNT for immunotherapy in *EGFR*-mutant lung cancer (Table 4). In the univariate analyses, histology, *EGFR* mutation type, sex, age, and smoking history were not associated with TTNT for ICIs. PS 0–1 at ICI initiation, ChemoIO, and treatment line (≤ third line) were positively correlated with the TTNT for immunotherapy. Multivariate analyses revealed that PS 0–1 at ICI initiation, minor *EGFR* mutations, and ChemoIO had a positive correlation with TTNT.

**Table 4:** Univariate and multivariate analyses of the factors associated with the TTNT of ICI treatment

	Univariate analyses		Multivariate analyses	
	HR (95% CI)	*p* value	HR (95% CI)	*p* value
PS at ICI initiation (2–4 vs. 0–1)	3.398 (1.776–6.502)	< 0.001	3.309 (1.712–6.395)	< 0.001
Histology (Sq vs. Ad)	0.377 (0.090–1.571)	0.181	0.281 (0.066–1.197)	0.086
*EGFR* mutation type (others vs. 19 del or L858R)	0.565 (0.265–1.202)	0.139	0.450 (0.205–0.987)	0.046
Treatment (ICI + Chemotherapy vs. ICI monotherapy)	0.487 (0.279–0.849)	0.011	0.389 (0.210–0.720)	0.003
Sex (male vs. female)	1.001 (0.603–1.663)	0.995	excluded	
Age (≥ 75 years vs. < 75 years)	1.180 (0.624–2.231)	0.608	excluded	
Smoking history (yes vs. no)	0.900 (0.536–1.511)	0.691	excluded	
Line of treatment (≥ fourth line vs. ≤ third line)	1.774 (1.055–2.982)	0.030	excluded	

*TTNT* time to next treatment, *ICI* immune checkpoint inhibitor, *HR* hazard ratio, *CI* confidence interval, *PS* performance status, *Ad* adenocarcinoma, *Sq* squamous cell carcinoma, *EGFR* epidermal growth factor receptor, *19 del* exon 19 deletion, *L858R* exon 21 L858R point mutation

Next, we examined the impact of these clinical factors on the OS of patients with *EGFR-*mutant lung cancer. Patients with PS 0–1 exhibited a superior OS than those with PS 2–4 (median 69.3 vs. 61.9 months, *p* = 0.005) (Supplementary Fig. 3a). Patients with the use of ChemoIO did not show a significant prolongation of OS compared to patients with ICI monotherapy (median: 63.3 vs. 49.0 months, *p* = 0.230) (Supplementary Fig. 3b). Patients with lung cancers harboring minor *EGFR* mutations showed a tendency of longer TTNT for ICIs (median 7.6 vs. 3.8 months, *p* = 0.133) than patients with major *EGFR* mutations. In contrast, the OS tended to be shorter in patients with minor *EGFR* mutations, but without statistical significance (median: 62.7 vs. 35.1 months, *p* = 0.636) (Supplementary Fig. 3c), again suggesting that the importance of EGFR-TKI duration for survival benefit in *EGFR*-mutated lung cancer (Supplementary Fig. 3d).

In our study, a long-term response (TTNT > 2 years) was observed in 4 patients (Table 5). All patients had a good PS (0–1). However, 3 of these 4 patients had a major *EGFR* mutation, and 3 of them were treated with ICI monotherapy. Additionally, ICI monotherapy was administered after 4th line therapy in 2 of the 3 patients. These results suggest the limitation of clinical characteristics to predict of long-term response induced by ICI in *EGFR*-mutant lung cancer.

**Table 5:** Characteristics of long term responder

Case	age	sex	PS	Histo-logy	Stage	*EGFR* mutation	Smoking	BI	Treatment of ICI	Treatment of TKI	Duration of TKI	Line of ICI	RT	response	irAE
1	68	M	1	Sq	Rec	19 del	current	960	Nivo	Erlotinib	240 days	4	-	SD	+
2	49	M	1	Ad	Rec	ex18 G719A	former	480	Nivo	Afatinib	330 days	6	+	unknown	+
3	84	F	0	Ad	IVB	19 del	former	150	CBDCA + PEM + Pemb	Afatinib	321 days	2	-	PR	+
4	63	F	0	Ad	IVB	L858R	never	0	Nivo	Erlotinib	57 days	3	+	PR	-

*M* male, *F* female, *PS* performance status, *Ad* adenocarcinoma, *Sq* squamous cell carcinoma, *Rec* recurrence, *EGFR* epidermal growth factor receptor; *19 del* exon 19 deletion, *ex18 G719A* exon 18 G719A mutation, *L858R* exon 21 L858R point mutation, *BI* Brinkman index, *ICI* immune checkpoint inhibitor; *Nivo* nivolumab, *CBDCA* carboplatin, *PEM* pemetrexed, *Pemb* Pembrolizumab, *TKI* tyrosine kinase inhibitor, *RT* radiation therapy, *SD* stable disease, *PR* partial response, *irAE* immune related adverse events

## Discussion

Few prospective observational studies have assessed the clinical characteristics of patients treated (ICI group) and not treated (Non-ICI group) with ICIs in *EGFR*-mutant lung cancer. This prospective observational study demonstrated that ICIs were administered to only 22% of patients, and they benefited less from initial use of EGFR-TKI regardless of 1st, 2nd or, 3rd generation, than those who were not treated with ICIs. It was thought that ICI might not yet be used because of the long duration of response to EGFR-TKI. In addition, the Non-ICI group did not show a significant prolongation of OS compared to the ICI group, but tended to have a superior OS. Previous studies revealed that a short duration of response to EGFR-TKIs correlated with benefit of ICI treatment in patients with *EGFR*-mutant lung cancers (Yoshida et al. 2018; Liu et al. 2021). However, our study revealed that the effect of ICI treatment does not neutralize the short duration of response to EGFR-TKIs, suggesting the importance of long-term response to EGFR-TKIs in terms of survival benefits in patients with *EGFR*-mutant lung cancer.

While the expected benefit of immunotherapy is limited in *EGFR*-mutant lung cancers, our study revealed that PS (0–1) was correlated with DCB of ICI therapies in *EGFR*-mutated lung cancers. PS is a well-known prognostic factor (Kawaguchi et al. 2010; Simmons et al. 2015). Therefore, it is challenging to evaluate the impact of PS on the efficacy of immunotherapy. Currently, multiple studies have reported the poor effect of ICI in patients with PS (Facchinetti et al. 2020; Miura et al. 2023). Given that these reports and the four cases of long-term response to immunotherapy (> 2 years) had good PS in our study, if treatment with ICIs is planned for *EGFR*-mutant lung cancer, it should be considered at least in patients who have maintained a good PS (0–1).

In this study, multivariate analysis showed that ChemoIO was a favorable factor for the prolongation of TTNT. Currently, several studies have failed to show the benefit of ChemoIO in *EGFR*-mutant lung cancers (Mok et al. 2022; Yang et al. 2023). In addition, ChemoIO with anti-angiogenic agents showed inconsistent and incompatible results in the same populations (Nogami et al. 2022; Zhou et al. 2023; Park et al. 2023). A subset analysis of the Impower150 trial indicated that ABCP may have a benefit on OS compared with bevacizumab plus carboplatin and paclitaxel chemotherapy in *EGFR*-mutant lung cancer (Nogami et al. 2022). This previous study may have prompted the administration of ABCP in patients with PS 0–1 in our cohort. However IMPOWER151 trial and ATTLAS trial failed to reproduce the benefit (Zhou et al. 2023; Park et al. 2023). Consistent with previous studies, our study also failed to show the significant prolongation of OS in the patients treated with ChemoIO compared with those without ChemoIO; thus, ChemoIO (including ABCP) is still not a standard treatment for *EGFR*-mutant lung cancer.

Consistent with previous reports (Yoshida et al. 2018), the TTNT of ICIs was superior in lung cancer with *EGFR* minor mutation than that with *EGFR* major mutation; however, the benefit in OS was inverse. This may be explained by the duration of administration of EGFR-TKIs because of the relatively worse effect of EGFR-TKIs in patients with *EGFR* minor mutation than in those with *EGFR* major mutation (Castellanos et al. 2017).

Patients with long-term response to immunotherapy (> 2 years) did not possess the clinical factors extracted by multivariate analysis in this study, suggesting a limitation in predicting the long-term response to immunotherapy using only clinical factors. A previous preclinical study revealed that oncogenic *EGFR* mutations play an important role in creating a non-inflamed tumor microenvironment (TME) (Nishii et al. 2022; Sugiyama et al. 2020) and that the expression of PD-L1 in cancer cells do not reproducibly predict the efficacy of ICI in *EGFR*-mutant lung cancer (Qiao et al. 2021). A previous report showed that cytotoxic T cells and the chemokines that recruit them are associated with the efficacy of ICIs in *EGFR-*mutant lung cancer (Hayashi et al. 2022), suggesting that some *EGFR*-lung cancers have an inflamed TME. Patients with lung cancer in the DCB group or long-term responders in our study may have such biological features. There is a strong need to establish biomarkers to identify effective populations for immunotherapy.

Our study has some limitations. First, eliminating registration and selection biases was difficult. Second, the number of factors for the multivariate analysis was limited because of the limited sample size. Third, data pertaining to PD-L1 expression, resistance mechanism to EGFR-TKI, or blood test results prior to ICI treatment were limited. In addition, the impact of co-occurring gene mutations such as TP53 mutations, which were reported to positively correlate with the effect with ICI (Dong et al. 2017; Sun et al. 2020), was not investigated in this study. Fourth, TTNT was used instead of PFS, which had a stronger correlation with OS, because it was difficult to obtain PFS for all the patients in this registry study. Therefore, we must carefully interpret our data for application in clinical practice. However, our study provides valuable real-world data regarding *EGFR*-mutant lung cancer.

In conclusion, our study demonstrates that patients treated with ICIs benefited less from EGFR-TKI treatment than those who were not treated with ICIs. In *EGFR*-lung cancer, it is difficult to predict the responder to ICI with OS prolongation based on clinical factors. Further studies to establish a biomarker based on the biological characteristics of *EGFR*-mutant lung cancers are warranted.

### Supplementary Information

Below is the link to the electronic supplementary material.

Supplementary file1 (TIF 552 KB)

Supplementary file2 (TIF 555 KB)

Supplementary file3 (TIF 558 KB)

Supplementary file4 (TIF 567 KB)

Supplementary file5 (TIF 547 KB)

Supplementary file6 (TIF 587 KB)

Supplementary file7 (TIF 553 KB)

Supplementary file8 (DOCX 18 KB)

Supplementary file9 (DOCX 17 KB)

Supplementary file10 (DOCX 17 KB)

## Data Availability

The datasets generated and/or analyzed during the current study are potentially available from the corresponding author on reasonable request.
