# Supplementary figures and images for: Clinical characteristics of patients treated with immune checkpoint inhibitors in EGFR-mutant non-small cell lung cancer: CS-Lung-003 prospective observational registry study
Source: J Cancer Res Clin Oncol. 2024 Feb 12;150(2):89. doi: 10.1007/s00432-024-05618-4 (PMC10861387; doi:10.1007/s00432-024-05618-4)

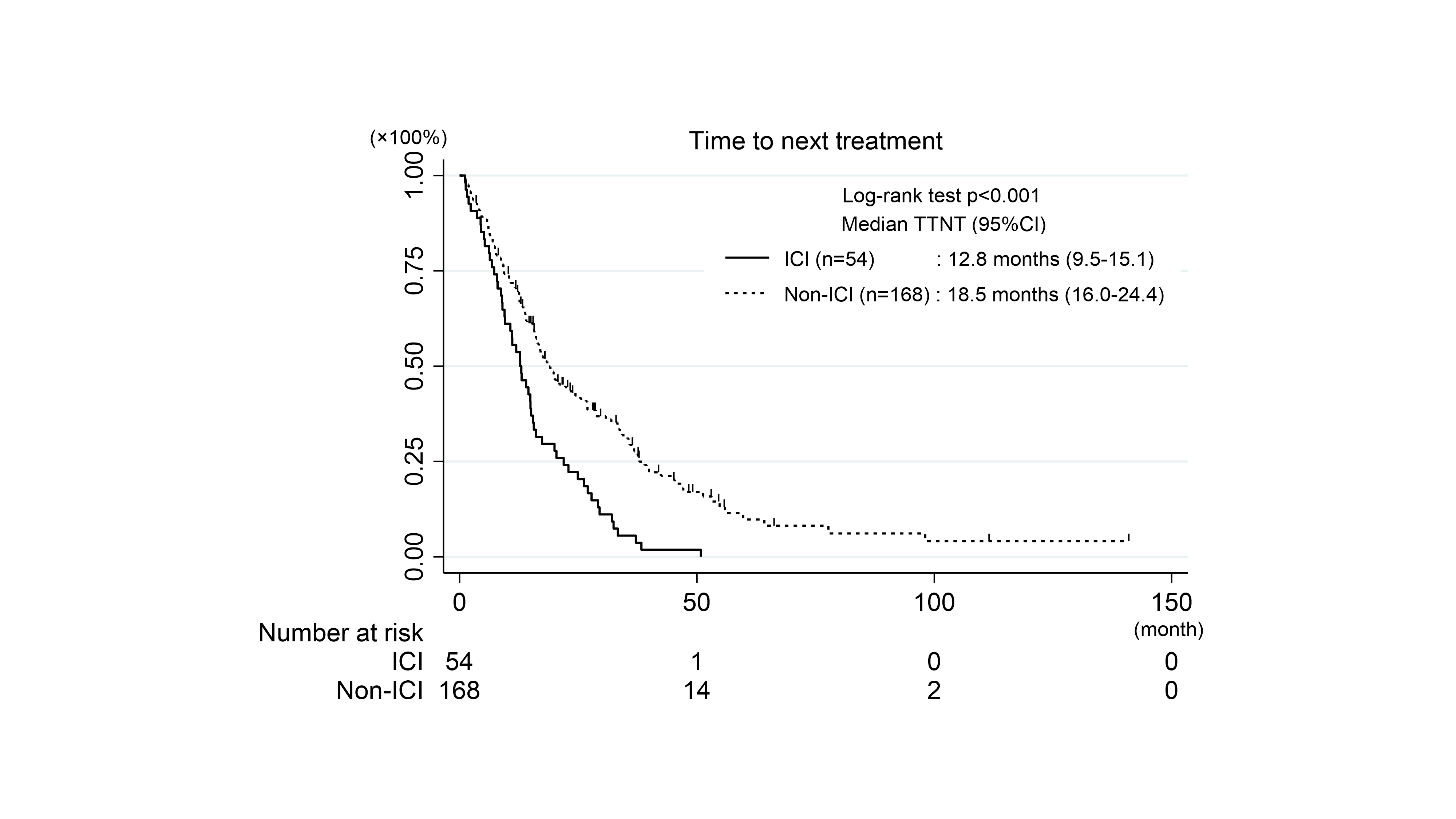

Supplement: Supplementary file 1 — Supplementary file1 (TIF 552 KB) [file 432_2024_5618_MOESM1_ESM.tif]

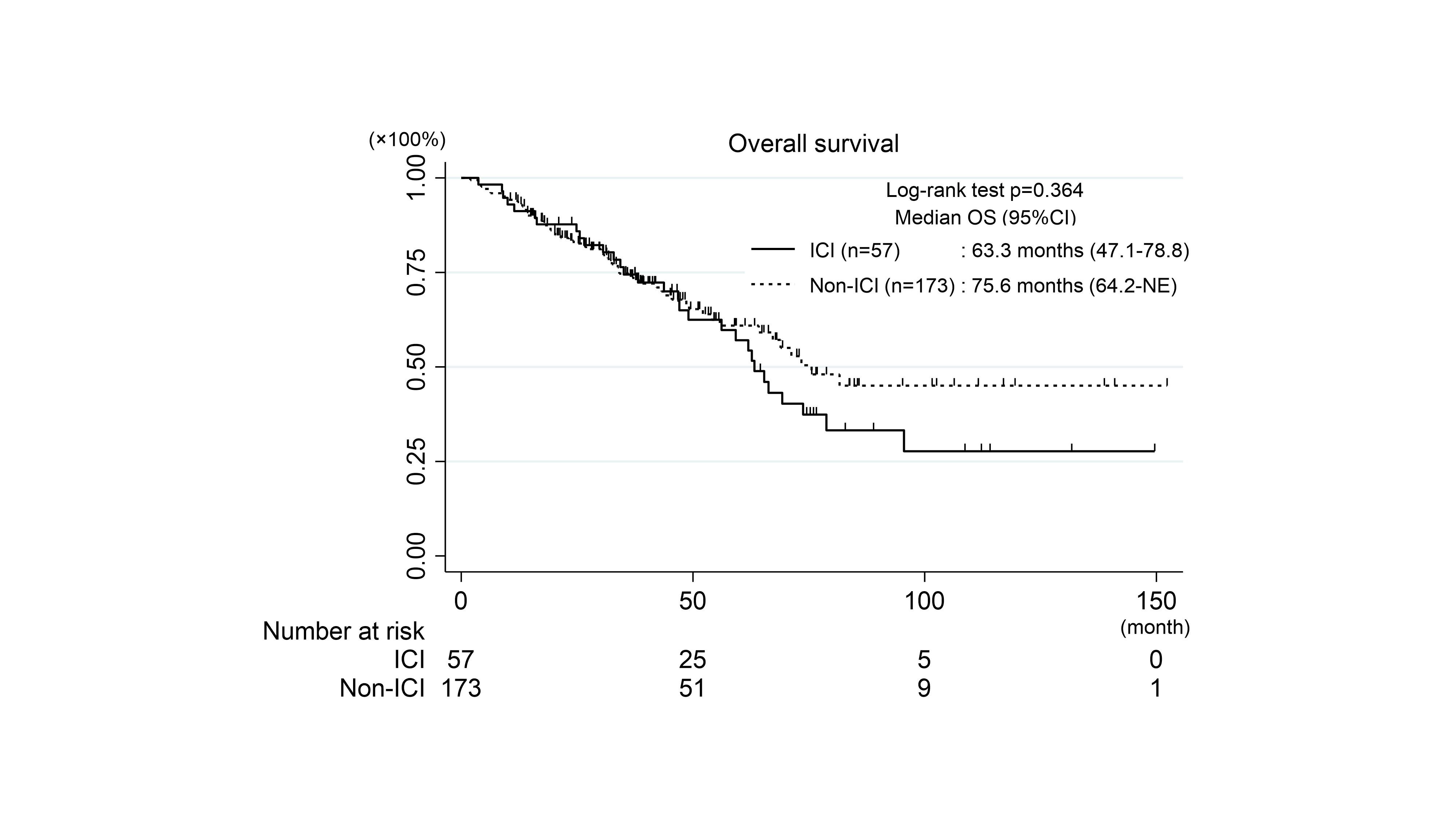

Supplement: Supplementary file 2 — Supplementary file2 (TIF 555 KB) [file 432_2024_5618_MOESM2_ESM.tif]

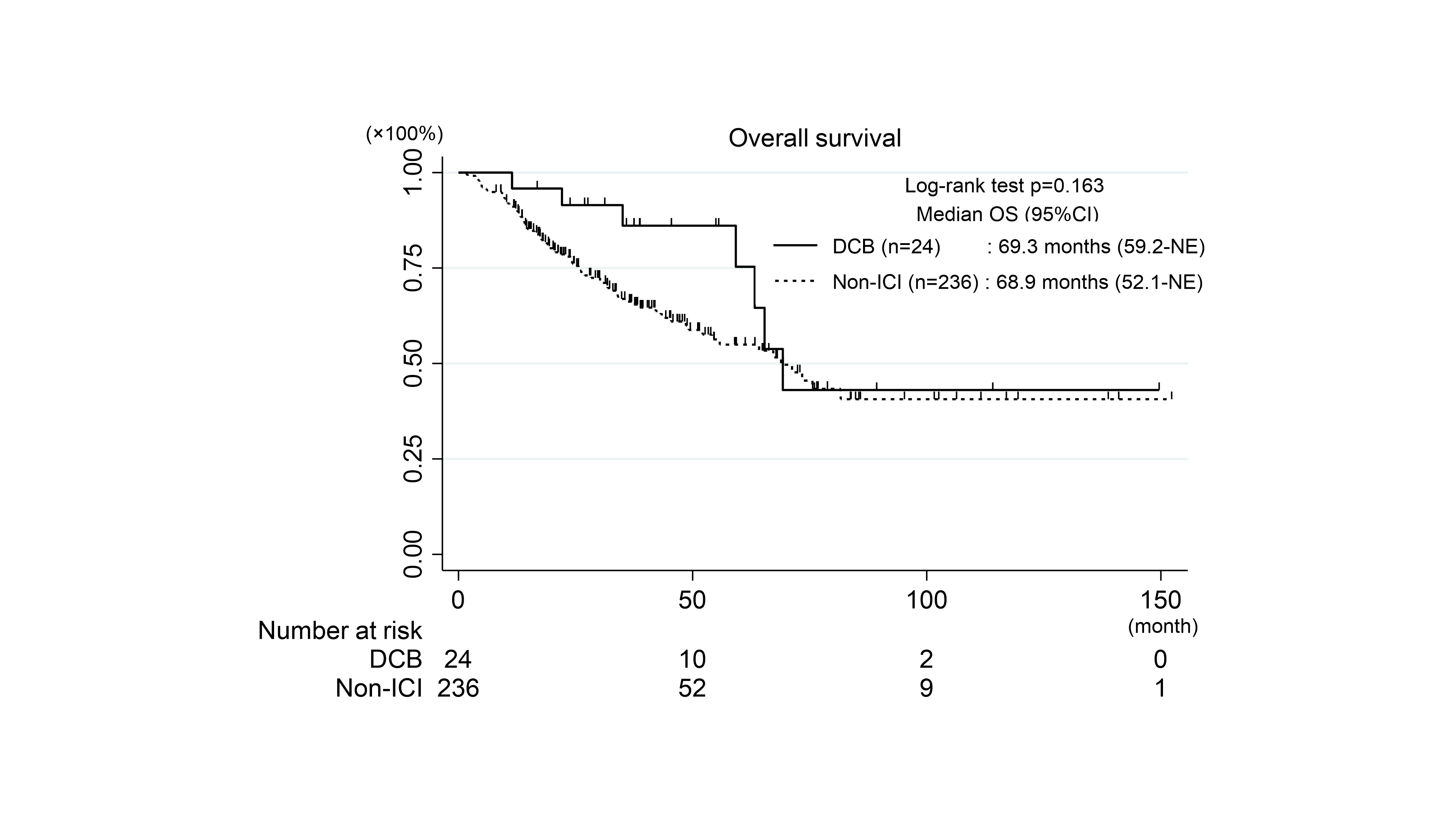

Supplement: Supplementary file 3 — Supplementary file3 (TIF 558 KB) [file 432_2024_5618_MOESM3_ESM.tif]

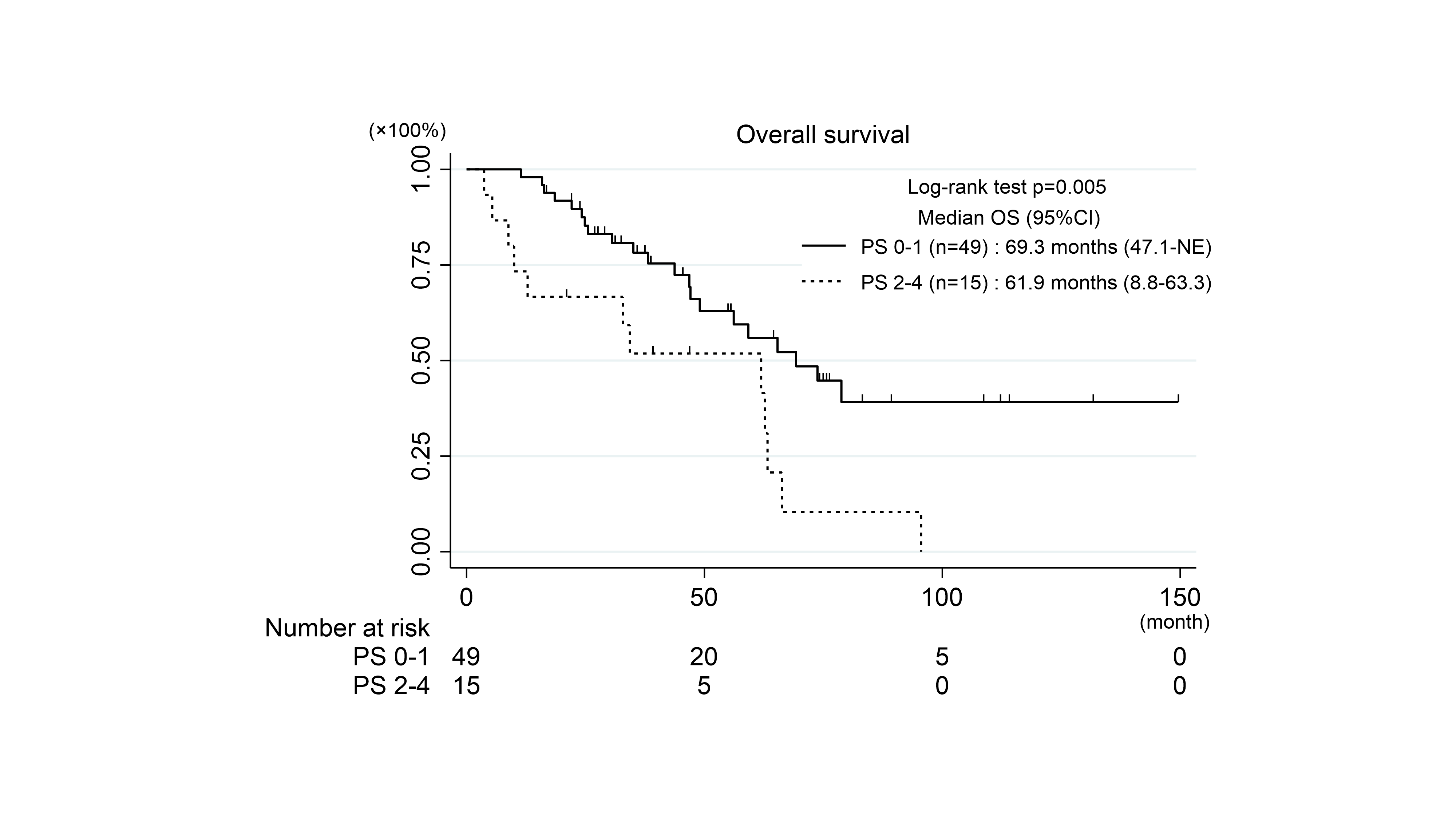

Supplement: Supplementary file 4 — Supplementary file4 (TIF 567 KB) [file 432_2024_5618_MOESM4_ESM.tif]

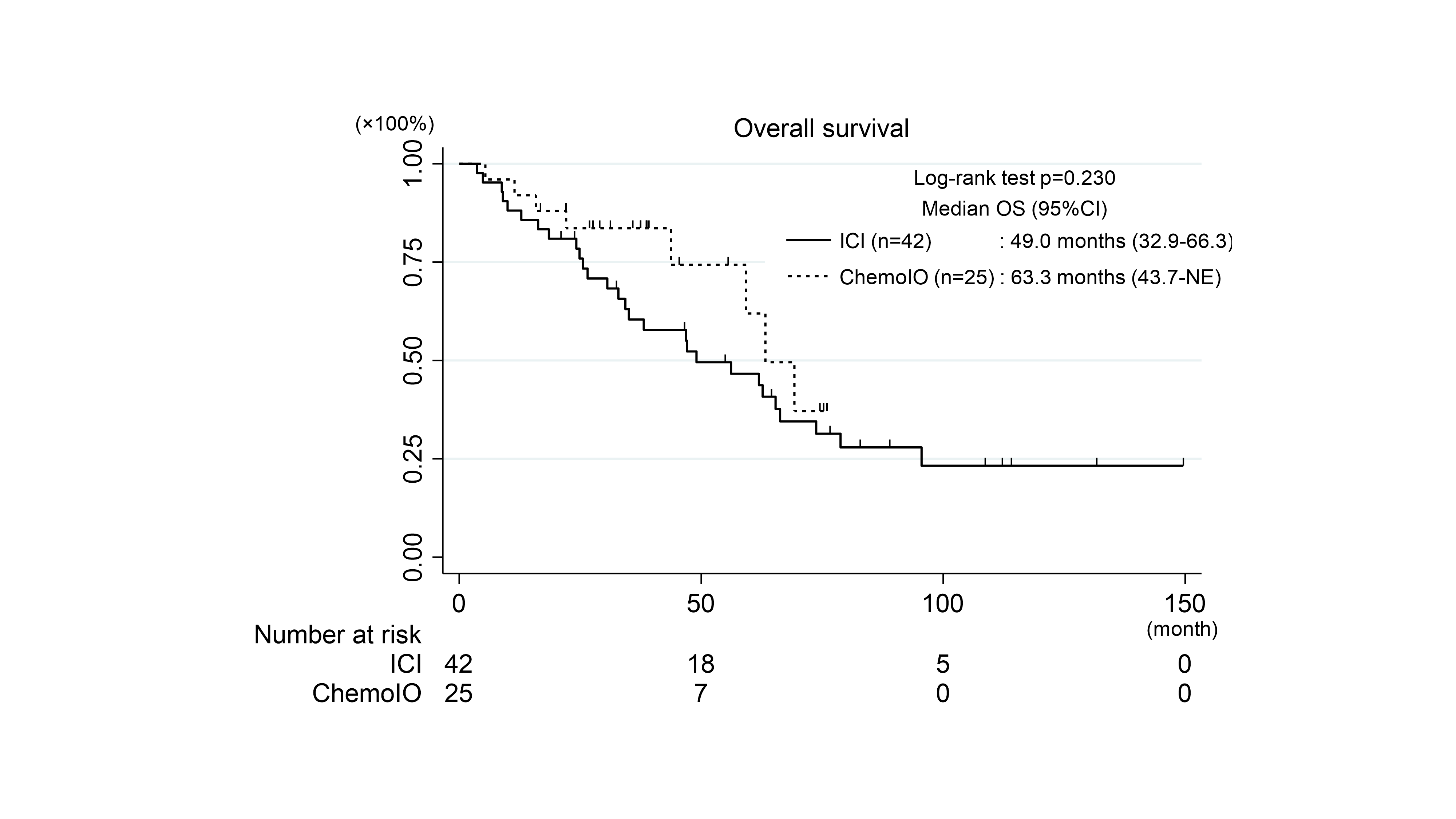

Supplement: Supplementary file 5 — Supplementary file5 (TIF 547 KB) [file 432_2024_5618_MOESM5_ESM.tif]

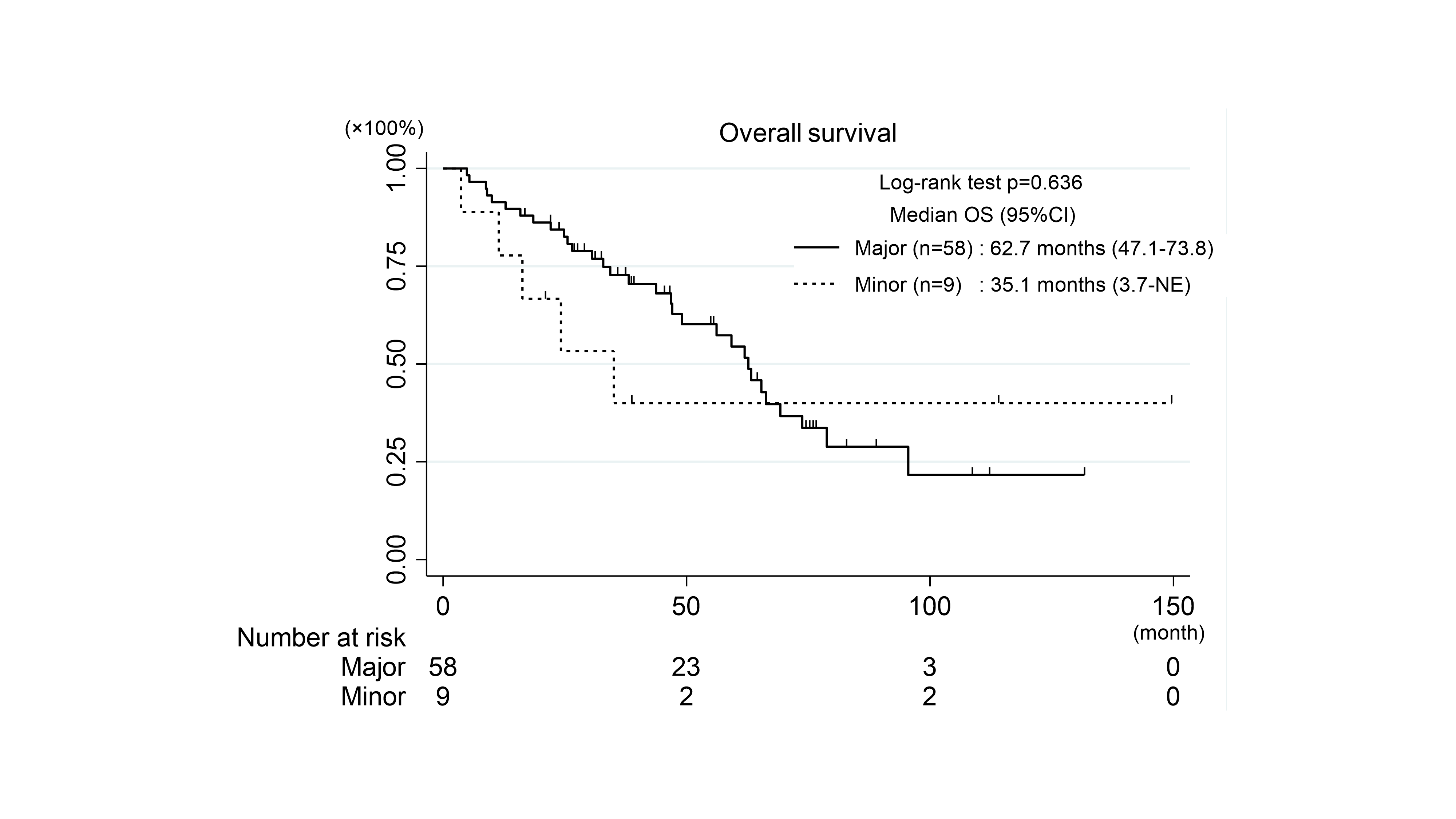

Supplement: Supplementary file 6 — Supplementary file6 (TIF 587 KB) [file 432_2024_5618_MOESM6_ESM.tif]

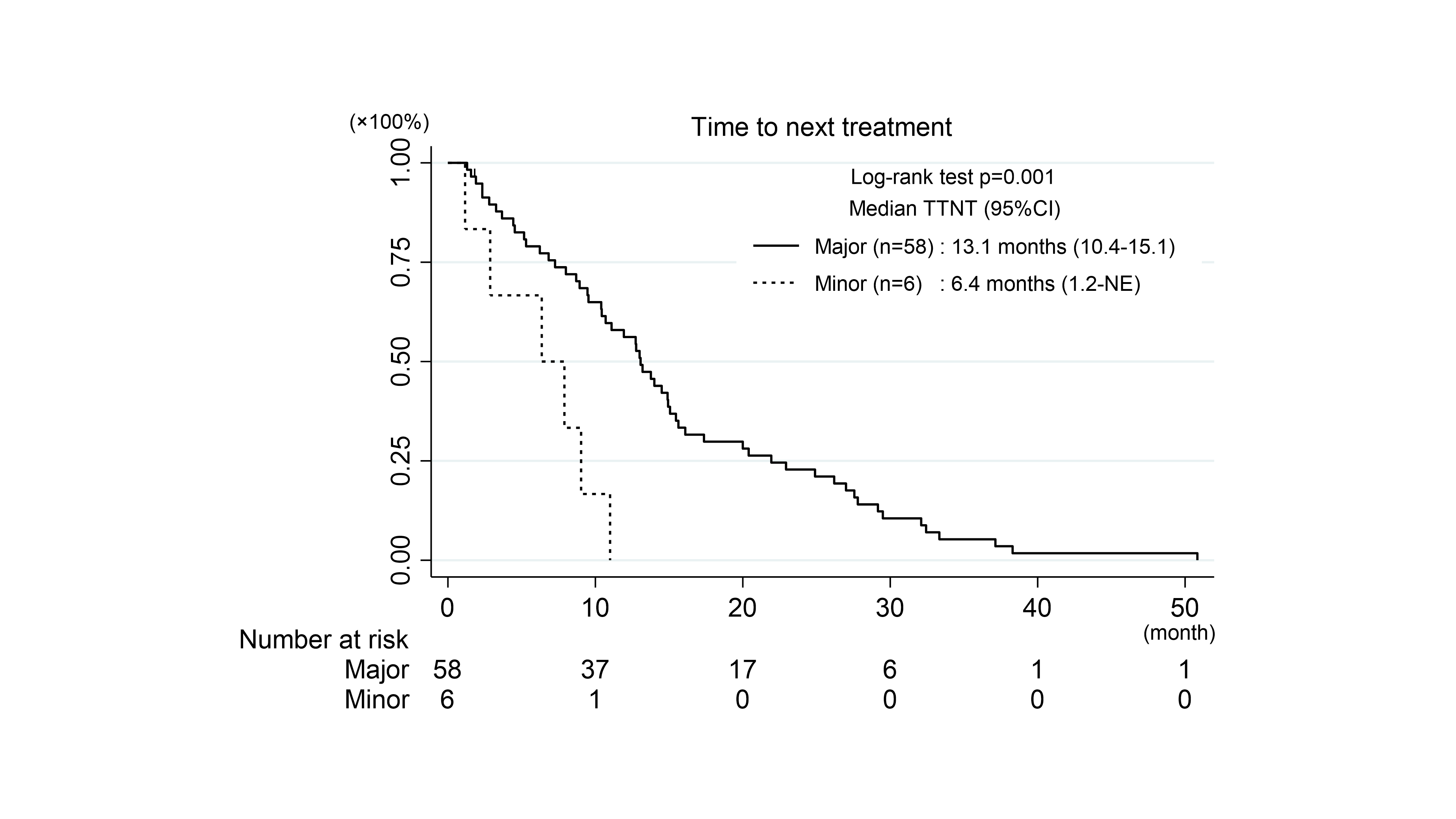

Supplement: Supplementary file 7 — Supplementary file7 (TIF 553 KB) [file 432_2024_5618_MOESM7_ESM.tif]
