# Supplementary material for: Clinical characteristics of patients treated with immune checkpoint inhibitors in EGFR-mutant non-small cell lung cancer: CS-Lung-003 prospective observational registry study
Source: J Cancer Res Clin Oncol. 2024 Feb 12;150(2):89. doi: 10.1007/s00432-024-05618-4 (PMC10861387; doi:10.1007/s00432-024-05618-4)
Supplement: Supplementary file 8 — Supplementary file8 (DOCX 18 KB) [file 432_2024_5618_MOESM8_ESM.docx]

| **Supplementary Table 1 Patient characteristics of ICI and Non-ICI groups without the patients treated with EGFR-TKI of 3^rd^ generations** | | | |
| --- | --- | --- | --- |
|  | ICI (n=57) | Non-ICI (n=173) | p value |
| Median age, years (range) | 64 (39-84) | 69 (36-98) |  |
| Age (≥75 years/<75 years) | 12 (21%)/45 (79%) | 51 (29%)/122 (71%) | 0.236 |
| Sex (male/female) | 28 (49%)/29 (51%) | 57 (33%)/116 (67%) | 0.039 |
| Stage (Ⅲ, Ⅳ/recurrent) | 40 (70%)/17 (30%) | 134 (77%)/39 (23%) | 0.288 |
| Histology (Ad/others) | 55 (96%)/2 (4%) | 168 (97%)/5 (3%) | 1.000 |
| PS at the initiation of systemic therapy (0-1/2-4) | 52 (91%)/2 (4%) | 145 (84%)/20 (12%) | 0.114 |
| *EGFR* mutation type (19 del or L858R/others) | 49 (86%)/8 (14%) | 164 (95%)/9 (5%) | 0.039 |
| Metastasis of brain (yes/no) | 6 (11%)/51 (89%) | 33 (19%)/140 (81%) | 0.158 |
| Metastasis of liver (yes/no) | 5 (9%)/52 (91%) | 13 (8%)/160 (92%) | 0.778 |
| Smoking history (yes/no) | 28 (49%)/27 (47%) | 60 (35%)/111 (64%) | 0.040 |
| Abbreviations: ICI, immune checkpoint inhibitor; TKI, tyrosine kinase inhibitor; Ad, adenocarcinoma; PS, performance status; EGFR, epidermal growth factor receptor; 19 del, exon 19 deletion; L858R, exon 21 L858R point mutation | | | |
