# Supplementary material for: Clinical characteristics of patients treated with immune checkpoint inhibitors in EGFR-mutant non-small cell lung cancer: CS-Lung-003 prospective observational registry study
Source: J Cancer Res Clin Oncol. 2024 Feb 12;150(2):89. doi: 10.1007/s00432-024-05618-4 (PMC10861387; doi:10.1007/s00432-024-05618-4)
Supplement: Supplementary file 9 — Supplementary file9 (DOCX 17 KB) [file 432_2024_5618_MOESM9_ESM.docx]

| **Supplementary Table 2 minor *EGFR* mutations of DCB and Non-DCB groups** | | |
| --- | --- | --- |
|  | DCB (n=5) | Non-DCB (n=4) |
| exon18 G719A | 1 | 1 |
| exon18 G719S | 1 | 1 |
| exon18 Del18 | 1 | 0 |
| exon20 T790M | 1 | 0 |
| exon20 H773dup | 0 | 1 |
| exon20 unknown | 0 | 1 |
| exon21 L861Q | 1 | 0 |
