# Supplementary material for: Clinical characteristics of patients treated with immune checkpoint inhibitors in EGFR-mutant non-small cell lung cancer: CS-Lung-003 prospective observational registry study
Source: J Cancer Res Clin Oncol. 2024 Feb 12;150(2):89. doi: 10.1007/s00432-024-05618-4 (PMC10861387; doi:10.1007/s00432-024-05618-4)
Supplement: Supplementary file 10 — Supplementary file10 (DOCX 17 KB) [file 432_2024_5618_MOESM10_ESM.docx]

# Supplementary Material

Supplementary Figure 1. Kaplan–Meier curves for TTNT (a) and OS (b) in patients without using of EGFR-TKI of the 3^rd^ generation.

Supplementary Figure 2. Kaplan–Meier curves for OS in patients with DCB and Non-ICI.

Supplementary Figure 3. Kaplan-Meier curves for OS (a) in patients with PS 0-1 and 2-4. Kaplan-Meier curves for OS (b) in patients with ChemoIO and ICI monotherapy. Kaplan-Meier curves for OS (c) and TTNT of EGFR-TKI (d) in patients with *EGFR* major mutation and minor mutation.

Abbreviations: TTNT, time to next treatment; OS, overall survival; EGFR, Epidermal growth factor receptor; TKI, tyrosine kinase inhibitor; DCB, durable clinical benefit; ICI, immune-checkpoint inhibitors; PS*,* performance status; ChemoIO, chemotherapy and ICIs combination; CI, confidence interval; NE, not evaluable
